# Condensed Tannins from Mangrove Species *Kandelia candel* and *Rhizophora mangle* and Their Antioxidant Activity

**DOI:** 10.3390/molecules15010420

**Published:** 2010-01-20

**Authors:** Liang-Liang Zhang, Yi-Ming Lin, Hai-Chao Zhou, Shu-Dong Wei, Jia-Hong Chen

**Affiliations:** 1 Key Laboratory of the Ministry of Education for Coastal and Wetland Ecosystems, School of Life Sciences, Xiamen University, Xiamen 361005, China; 2 Key and Open Lab. on Forest Chemical Engineering SFA, Institute of Chemical Industry of Forest Products, CAF; Nanjing 210042, China

**Keywords:** mangrove, condensed tannins, free radical scavenging activity, reducing power

## Abstract

The structures of condensed tannins isolated from two mangrove species, *Kandelia candel* and *Rhizophora mangle,* were characterized by ^13^C nuclear magnetic resonance (NMR) spectroscopy and matrix assisted laser desorption/ionization time-of-flight mass spectrometry (MALDI-TOF MS) analyses. Results demonstrate that large heterogeneity occurs in degree of polymerization, pattern of hydroxylation, and substitution with monosaccharides in the structures of the condensed tannins. Condensed tannin oligomers from *K. candel* and *R. mangle* were shown to be heterogeneous mixtures consisting of procyanidin and prodelphinidin structural units with the former dominating. The MALDI-TOF mass spectra contained masses corresponding to a distinct oligomeric series of glycosylated heteropolyﬂavan units. In addition, condensed tannins from two mangrove plants were screened for their potential antioxidant activities using 1,1-diphenyl-2-picrylhydrazyl (DPPH) and ferric reducing antioxidant power (FRAP) model systems.

## 1. Introduction

Condensed tannins comprise a group of polyhydroxyflavan-3-ol oligomers and polymers linked by carbon-carbon bonds between flavanol subunits [1]. The most common classes are the procyanidins, which are chains of catechin, epicatechin, and their gallic acid esters, and the prodelphinidins, which consist of gallocatechin, epigallocatechin, and their galloylated derivatives as the monomeric units [2]. The properties of condensed tannins depend on their structures in terms of monomer units, their mean degree of polymerization (DP) and the linkage-type between ﬂavan-3-ol units with a considerable range of structural variation [3]. Condensed tannins have attracted great attention due to rapid growing evidence associating these compounds with a wide range of potential health benefits. Recently, it has been suggested that any potential health benefits attributed to these compounds may be affected by the degree of polymerization [4]. However, detailed information on the condensed tannin profiles present in most plants is currently lacking, especially with regard to the more complex oligomeric structures, and analysis of higher condensed tannins is not feasible, since the number of isomers increases with increasing degrees of polymerization.

In mangrove species, condensed tannins are abundant components (as high as 20% dry weight) which prevent damage from herbivores [5,6], but they also show a diversity of other biological activities of historic and potential importance to humans [7]. In addition to application in leather tanning, mangrove extracts have been used for diverse medicinal purposes and have a variety of antibacterial, antiherpetic and antihelminthic activities [8,9]. The extracts of some mangrove species indicated significant antioxidant activity [10,11], and we supposed the active compounds responsible for antioxidant activity were tannins. The structures of the main monomers constituting the condensed tannins were identiﬁed, these being catechin, epicatechin, epigallocatechin, and epicatechin gallate [12]. However, different condensed tannins present different structures and different degrees of polymerization [13] and these are unknown in the case of mangrove condensed tannins.

Because of the complex structural diversity and related physiochemical properties, condensed tannins were considered to be the final frontier of ﬂavonoid research [14], and unexplored condensed tannins from mangrove plants may be potential resources for novel bioactive compounds. This study was designed to separate and characterize condensed tannins from two mangrove species (*Kandelia candel* and *Rhizophora mangle*), and to investigate their antioxidant activities. The isolated condensed tannins were subjected to ^13^C-NMR spectroscopy and matrix assisted laser desorption/ionization time-of-flight mass spectrometry (MALDI-TOF MS), which allows the detection of molecules with high molecular masses (from several hundred to several thousand Daltons). Due to the gentle ionization, fragmentation of the macromolecule under research can be avoided. Meanwhile, the free radical scavenging capacities and ferric reducing power of condensed tannins from two mangrove species were also discussed.

## 2. Results and Discussion

### 2.1. Content of total phenolics and extractable condensed tannins

Total phenolics contents in the leaves of *K. candel* and *R. mangle* were 130.32 ± 4.66 mg/g and 182.62 ± 21.43 mg/g, respectively (Table 1). Extractable condensed tannin contents in the leaves of *K. candel* and *R. mangle* were 106.35 ± 21.16 mg/g and 219.27 ± 63.11 mg/g, respectively. Polyphenols are the major plant compounds with antioxidant activity. The results strongly suggest that phenolics are important components in these plants, and some of their pharmacological effects could be attributed to the presence of these valuable constituents.

**Table 1:** The contents of total phenolics (TP) and extractable condensed tannins (ECT) from *K. candel* and *R. mangle*.

Samples	TP (mg/g) *^a^*	ECT (mg/g) *^b^*
*K. candel*	130.32 ± 4.66b	106.35 ± 21.16b
*R. mangle*	182.62 ± 21.43a	219.27 ± 63.11a

***^a^***TP were expressed as tannic acid as the standard; ***^b^*** ECT were expressed as purified mangrove condensed tannins as the standard. Data are presented as the mean ± standard deviation (n = 3). Means with different letters are significant differences at *P* < 0.05 levels.

### 2.2. ^13^C-NMR analysis

**Figure 1:**
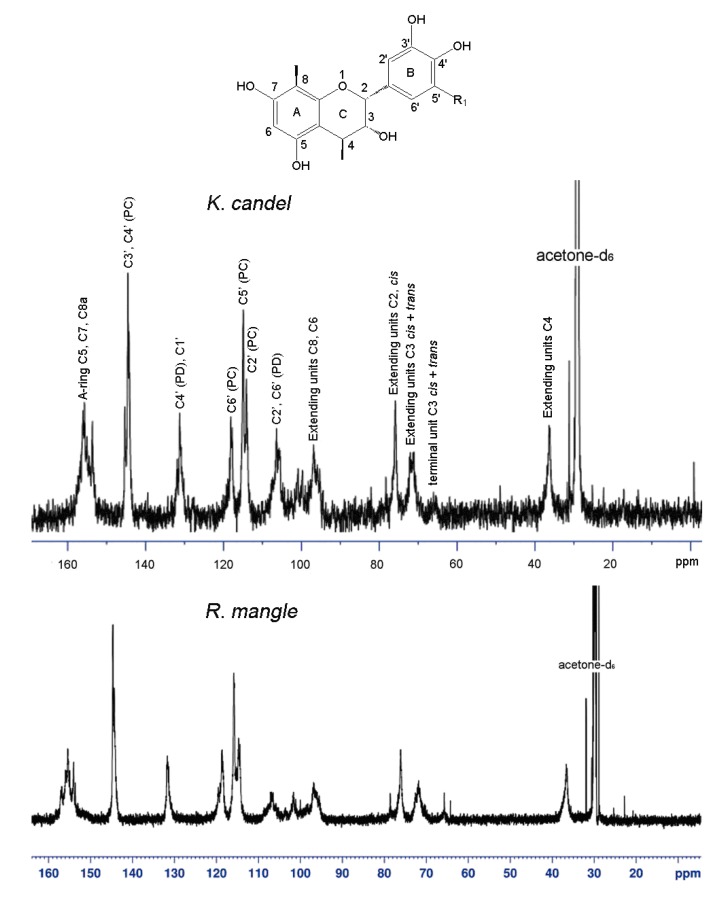
^13^C-NMR spectra (150 MHz) of condensed tannins from *K*. *candel* and *R. mangle*; solvent, acetone-*d_6_*/D_2_O; room temperature. Identity of the structures: R_1_=H, epicatechin; and R_1_=OH, epigallocatechin.

The condensed tannins were analyzed by ^13^C-NMR spectroscopy, and the signal assignment was performed according to the method by Czochanska *et al*. [15]. The ^13^C-NMR spectrum (Figure 1) of the *K. candel* condensed tannins in acetone-*d_6_*/D_2_O shows characteristic peaks consistent with those of condensed tannins with dominant procyanidin units and a minor amount of prodelphinidins. The structural diversity of the linkages (A and B type) and stereoisomers of catechin and epicatechin units is apparent from the spectrum. Specially, C5, C7, and C8a carbons of procyanidins appear at 150 to 160 ppm. Peaks at 145.2 and 145.3 ppm belong to C3′ and C4′ of procyanidin units. The signals at 114.8, 115.3 and 118.1 ppm are assignable to the C2′, C5′ and C6′ of procyanidin units. A small amount of prodelphinidins is also detected as its C4′ peak and appears at 131.2 ppm, overlapping with the chemical shifts of C1′. The cluster of peaks between 95 and 100 ppm is assigned to C8 and C6 of procyanidins, and the peaks between 104 and 108 ppm is assigned to C6′ and C2′ of prodelphinidins. The region between 70 and 90 ppm is sensitive to the stereochemistry of the C-ring. The ratio of the 2,3-*cis* to 2,3-*trans* isomers could be determined through the distinct differences in their respective C2 chemical shifts. C2 gives a resonance at 75.7 ppm for the *cis* form and 79 ppm for the *trans* form. From the peak areas, it is estimated that the *cis* isomer is dominant. C3 of both *cis* and *trans* isomers occurs at 71 ~ 72 ppm. The peak at 64 ppm is due to C3 of the terminal unit. The C4 atoms of the extension units showed a broad peak at 36 ppm, while the terminal C4 exhibits multiple lines at 29 and 27 ppm [16]. In addition, the glycosides were also detected in the spectrum. The carbon signals at 101.8 ppm (C’’1), 75～77 ppm (C’’2, C’’3 and C’’5), 71 ppm (C’’4), and 61 ppm (C’’6) were in agreement with those of the glycosides [17]. The sugar moiety was supported by the result of a sugar analysis of the acidic hydrolysis of mangrove condensed tannins. The carbon signal at 71.8 ppm (C3) suggested the glycose moiety to be connected to the C3 position. The ^13^C-NMR spectrum of condensed tannins from *R. mangle* was similar to that of *K. candel*. The typical signals due to procyanidin and prodelphinidin units with some glycosides were also found in *R. mangle* condensed tannins.

### 2.3. MALDI-TOF MS analysis

**Figure 2:**
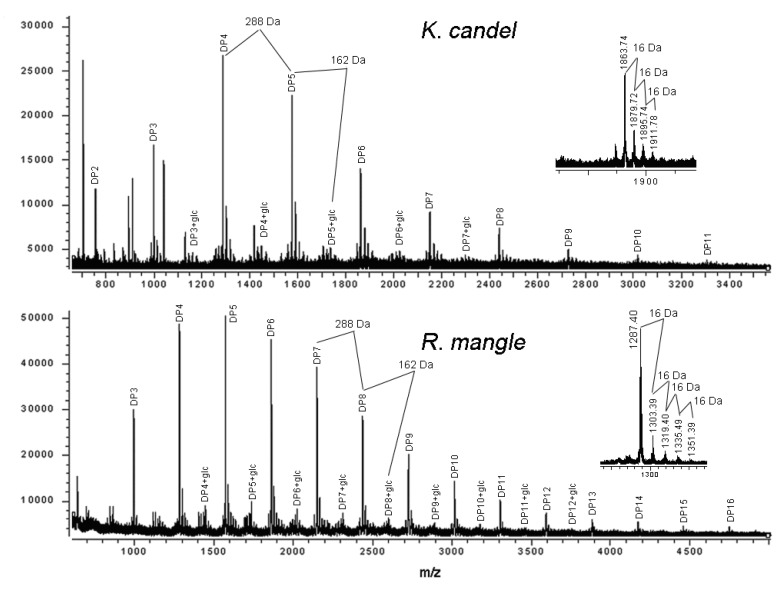
MALDI-TOF positive reflectron mode mass spectra of the condensed tannins from *K*. *candel* and *R. mangle*. Inset is an enlarged spectrum of masses representing a procyanidin series with varying hydroxylation patterns.

Although the ^13^C-NMR spectra revealed the complex structural characteristics of the mangrove condensed tannins, quantitative data regarding the degree of polymerization cannot be reliably obtained. Further characterization was achieved by MALDI-TOF mass spectrometry. Figure 2 shows the MALDI-TOF mass spectra of the condensed tannins isolated from *K*. *candel* and *R. mangle* leaves, recorded as Cs^+^ adducts in the positive ion reflectron mode and showing a series of repeating procyanidin polymers. The polymeric character is reflected by the periodic peak series representing different chain lengths. The masses of the highest peaks among the polyflavonoid tannin polymers from mangrove species with identical DP increased at the distance of 288 Da, corresponding to one catechin/epicatechin monomer (Table 2). Therefore, prolongation of condensed tannins is due to the addition of catechin/epicatechin monomers. Condensed tannins with a DP of 2 to 11 (*m/z* 711.33～3304.45) were detected in *K*. *candel*, and condensed tannins with a DP of 3 to 16 (*m/z* 999.34～4751.54) were detected in *R. mangle*. The spectra did not contain ions with 2 Da lower than that of the highest peaks among the polyflavan-3-ols polymers.

**Table 2:** Summary of peaks with the highest intensities in MALDI-TOF MS of the condensed tannins from *K*. *candel* and *R. mangle*.

Polymer	n_1_	n_2_	n_3_	*K. candel*	*R. mangle*
DP2	2	0	0	711.33	
DP3	3	0	0	999.35	999.34
	3	0	1	1161.35	
	2	1	0	1015.37	1015.34
	1	2	0	1031.38	
DP4	4	0	0	1287.43	1287.40
	4	0	1	1449.44	1449.46
	3	1	0	1303.42	1303.39
	3	1	1		1465.62
	2	2	0	1319.41	1319.40
	2	2	1		1481.58
	1	3	0		1335.49
	0	4	0		1351.39
DP5	5	0	0	1575.57	1575.51
	5	0	1	1737.71	1737.66
	4	1	0	1591.59	1591.51
	4	1	1		1753.77
	3	2	0	1607.56	1607.51
	3	2	1		1769.46
	2	3	0		1623.47
	1	4	0		1639.48
	0	5	0		1655.41
DP6	6	0	0	1863.74	1863.70
	6	0	1	2025.87	2026.83
	5	1	0	1879.72	1879.70
	5	1	1		2043.82
	4	2	0	1895.74	1895.70
	4	2	1		2058.75
	3	3	0	1911.78	1911.61
	2	4	0		1927.68
	1	5	0		1943.63
DP7	7	0	0	2151.97	2151.94
	7	0	1	2314.98	2314.24
	6	1	0	2167.97	2167.94
	5	2	0	2183.99	2183.99
	4	3	0		2199.95
	3	4	0		2215.94
DP8	8	0	0	2439.39	2440.30
	8	0	1		2603.31
	7	1	0	2456.38	2457.24
	6	2	0		2473.26
DP9	9	0	0	2727.79	2729.69
	9	0	1		2892.88
	8	1	0	2744.93	2745.69
	8	1	1		3018.09
DP10	10	0	0	3016.09	3017.09
	10	0	1		3180.47
	9	1	0		3033.95
	8	2	0		3050.11
DP11	11	0	0	3304.45	3307.23
	11	0	1		3469.28
	10	1	0		3323.31
DP12	12	0	0		3596.28
	12	0	1		3758.37
	11	1	0		3611.74
DP13	13	0	0		3884.89
	12	1	0		3899.78
DP14	14	0	0		4173.27
DP15	15	0	0		4463.13
DP16	16	0	0		4751.54

*n*_1_: Number of catechin unit; *n*_2_: Number of gallocatechin unit; *n*_3_: Number of glycoside.

In addition to the predicted homopolyflavan-3-ol mass series mentioned above, each DP had a subset of masses 16, 32 and 48 Da higher (Figure 2 and Table 2). These masses can be explained by heteropolymers of repeating flavan-3-ol units containing an additional hydroxyl group (∆16 Da) at the position 5' of the B-ring. Given the absolute masses corresponding to each peak, it was further suggested that they contain procyanidins and prodelphinidins, as have already been indicated in the ^13^C-NMR spectra. Each peak of the condensed tannins from studied mangrove species was always followed by mass signals at a distance of 162 Da corresponding to the addition of one glycoside group at the heterocyclic C-ring. Thus, peak signals corresponding to glycosylated derivatives of various procyanidin oligomers were easily attributed. No procyanidins with more than one glycoside group were detected. Therefore, MALDI-TOF mass spectrometry indicated the simultaneous occurrence of a mixture of procyanidin polymers and monoglycosylated derivatives of procyanidin polymers, showing that there was a mixture of glycosylated procyanidins and procyanidins occurring in condensed tannin oligomers from *K*. *candel* and *R. mangle* leaves (Figure 2). These were consistent with the evidence found in ^13^C-NMR spectra. A previous study has identified two flavan-3-ol glycosides from stems of the mangrove plant *R. stylosa* [18]. The MALDI-TOF mass spectrometry has demonstrated the potential to rapidly analyze flavonol glycosides in a complex extract. The series of compounds with 2 Da multiples lower than those described peaks for heteropolyflavan-3-ols were not detected, so A-type interflavan ether linkage does not occur between adjacent flavan-3-ol subunits. The galloyl group which showed the mass signals at a distance of 152 Da was not detected in the mass spectra although it has been reported in another mangrove species *R. apiculata* [19]. For the first time, compositional analysis of condensed tannins from mangrove species using MALDI-TOF MS and ^13^C-NMR have been successfully demonstrated.

The characterization of the condensed tannins was limited with respect to the detection of high mass condensed tannins and by the fact that peak intensities were not related to their concentration in the sample. The quality of the spectrum depended upon parameters such as the tannin/matrix ratio, the target area of the laser, and the crystallization of the mixture on the target. Therefore, it was very difficult to obtain a reproducible spectrum. The main problem is to find a new technique that permits the complete characterization of condensed tannins up to 4,000 Da, which allows an equal sensitivity for both low and high mass polymers.

### 2.4. Free radical scavenging activity

**Figure 3:**
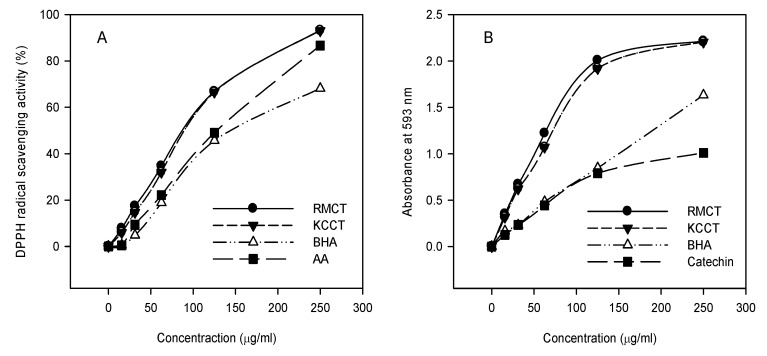
DPPH radical scavenging activity (A) and reducing power (B) of RMCT, KCCT and reference standards at different concentration. RMCT and KCCT were respective condensed tannins from *R*. *mangle* and *K*. *candel*.

The relatively stable organic radical DPPH has been widely used in the determination of antioxidant activity of single compounds as well as the different plant extracts [20,21,22,23,24,25]. The reduction capacity of DPPH was determined by the decrease in its absorbance at 517 nm, which is reduced by antioxidants [20]. The free radical scavenging activities of condensed tannins from *K*. *candel* and *R. mangle* along with the reference standards of ascorbic acid and BHA were determined by the DPPH assay. Because activities are expressed as the condensed tannins concentration required to achieving a 50% decrease in absorbance at 517 nm (IC_50_), the smaller condensed tannins concentration indicates the higher DPPH radical scavenging activity. Condensed tannins from two mangrove species all showed the significantly higher inhibition percent of DPPH radical compared to reference ascorbic acid and BHA. The IC_50_ values of condensed tannins from *R. mangle* and *K. candel* were 89.83 ± 4.91 µg/mL and 93.51 ± 4.44 µg/mL respectively (*P* > 0.05). It was reported that the glycoside moiety in condensed tannins structure can enhance the effectiveness of condensed tannins radical scavenging [18]. Figure 3A shows the DPPH free radical scavenging activity of the studied condensed tannins and the references at different concentrations, and demonstrated that all of the tested antioxidants showed dose-dependent activity. The free radical scavenging activity increased with the increasing concentration of condensed tannins.

### 2.5. Reducing antioxidant power

The reduction capacity of a compound may serve as a significant indicator of its potential antioxidant activity [26]. A higher absorbance corresponds to a higher ferric reducing power. For each antioxidant, different concentrations (0.01-0.5 mg/mL) were prepared. All condensed tannins showed increased ferric reducing power with the increasing concentration (0-0.5 mg/mL) (Figure 3B). In accordance with the findings from the DPPH assay, the reducing power of the condensed tannins from *R. mangle* (12.98 ± 1.20 mmol AAE/g) was superior to that of *K. candel* (10.77 ± 0.37 mmol AAE/g), BHA (4.19 ± 0.11 mmol AAE/g) and (3.89 ± 0.12 mmol AAE/g).

## 3. Experimental

### 3.1. Chemicals and plant materials

All solvents used were of analytical reagent (AR) purity grade. Ascorbic acid (AA), butylated hydroxyanisole (BHA), tannic acid, catechin, TPTZ (2,4,6-tripyridyl-S-triazine) and DPPH (1,1-diphenyl-2-picrylhydrazyl) were purchased from Sigma-Aldrich (USA). Sephadex LH-20 was purchased from Amersham (USA). The mangrove species *K. candel* and *R. mangle* were collected from a mangrove forest in the Dongzhai harbor (19°56′N, 110°34′E), Hainan, China. *R. mangle* was introduced from La Paz and Mazatlan City, Mexico in 1999 [27].

### 3.2. Extraction and purification of condensed tannins

Leaf samples were taken to the laboratory immediately after collection and cleaned with distilled water. The condensed tannins were extracted and purified as described by Lin *et al*. [28]. The condensed tannins were freeze-dried and stored at −20 °C before analysis by ^13^C-NMR and MALDI-TOF mass spectrometry.

### 3.3. Determination of total phenolics and extractable condensed tannins

Total phenolics and extractable condensed tannins of raw extracts of the leaves were determined. Total phenolics were measured with the Prussian Blue method [29], using tannic acid as the standard. Extractable condensed tannins were assayed by the butanol-HCl method [30], using purified condensed tannins from respective mangrove species as the standards.

### 3.4. ^13^C-NMR analysis

The ^13^C-NMR spectra of condensed tannins were recorded on Varian Metcury-600 spectrometer (USA) at 150 MHz. The samples for recording NMR spectra were prepared by dissolving the samples in acetone-*d_6_*/D_2_O mixture.

### 3.5. MALDI-TOF MS analysis

The MALDI-TOF mass spectra were recorded on a Bruker Reflex Ⅲ MALDI-TOF mass spectrometer (Germany). The irradiation source was a pulsed nitrogen laser with a wavelength of 337 nm, and the duration of the laser pulse was 3 ns. The measurements were carried out using the following conditions: in the positive ion mode, an accelerating voltage of 20.0 kV and a reflectron voltage of 23.0 kV were used. Cesium chloride and 2,5-dihydroxybenzoic acid (DHB) as the matrix were used to enhance ion formation. Amberlite IRP-64 cation-exchange resin (Sigma-Aldrich), equilibrated in deionized water, was used to deionize the analyte/matrix solution thrice. An aqueous solution of cesium chloride (0.5 µL, 1 mg/mL) was added to the sample solution (1.5 µL, 10 mg/mL aqueous) followed by addition of an equal volume of DHB (10 mg/mL aqueous solution). The resulting solution (1.0 µL) was evaporated and introduced into the mass spectrometer [31].

### 3.6. DPPH radical scavenging activity

To assess the scavenging ability on DPPH radicals, condensed tannins (0.1 mL, 15–250 µg/mL) in methanol were mixed with 3 mL of methanol solution containing DPPH radicals (0.004%, w/w). The mixture was shaken vigorously and left to stand for 30 min in the dark before measuring the absorbance at 517 nm against a blank [22]. Ascorbic acid and BHA were used as reference standards. Then the scavenging ability was calculated as: 

Scavenging ability (%) = [(∆*A*_517_ of control – ∆*A*_517_ of sample)/∆*A*_517_ of control] × 100

### 3.7. Reducing antioxidant power effects

Reducing power antioxidant effects were determined following the method by Benzie and Strain [32]. Briefly, FRAP reagent (3 mL), prepared freshly, was mixed with test sample (0.1 mL), or methanol (for the reagent blank). The FRAP reagent contained 10 mM TPTZ solution (2.5 mL) in 40 mM HCl plus 20 mM FeCl_3_ (2.5 mL) and 0.3 M acetate buffer, pH 3.6 (25 mL). The absorbance of reaction mixture was measured spectrophotometrically at 593 nm after incubation at 25 °C for 10 min. The FRAP values, expressed in mmol ascorbic acid equivalent (AAE)/g sample in dry weight were derived from a standard curve, and BHA and catechin were used as reference standards.

### 3.8. Statistical analysis

All measurements were replicated three times and a one-way ANOVA test was performed on the antioxidant activity results to investigate significant differences between two mangrove species. The method used to discriminate among the means was Duncan’s multiple range tests. The computer program employed was SPSS 11.0 for Windows.

## 4. Conclusions

Structures of condensed tannins from two mangrove species, *R. mangle* and *K. candel* characterized by ^13^C-NMR and MALDI-TOF MS analyses showed that the condensed tannins consisted of predominantly procyanidins with 2,3-cis stereochemistry. There are some of monoglycoside flavan-3-ols polymers with polymerization degree up to 12 were detected in mass spectra of mangrove species. Condensed tannins extracted from two mangrove species showed very good DPPH radical scavenging activity and ferric reducing power.
